# Spasticity-Plus syndrome in multiple sclerosis patients in a tertiary hospital in Spain

**DOI:** 10.3389/fneur.2024.1360032

**Published:** 2024-02-26

**Authors:** Haydee Goicochea Briceño, Yolanda Higueras, Irene Ruiz Pérez, José Manuel García Domínguez, Juan Pablo Cuello, Ariana Meldaña Rivera, María Luisa Martínez Ginés

**Affiliations:** ^1^Departamento de Enfermería, Hospital General Universitario Gregorio Marañón, Madrid, Spain; ^2^Facultad de Psicología, Universidad Complutense de Madrid, Madrid, Spain; ^3^Instituto de investigación Sanitaria Gregorio Marañón (IiSGM), Hospital General Universitario Gregorio Marañón, Madrid, Spain; ^4^Servicio de Neurología, Hospital General Universitario Gregorio Marañón, Madrid, Spain

**Keywords:** multiple sclerosis, spasticity, Spasticity-Plus syndrome, pain, nabiximols

## Abstract

**Introduction:**

Spasticity is a common symptom in multiple sclerosis (MS) and it is often associated with other symptoms such as spasms/cramps and pain. The concept of Spasticity-Plus syndrome takes into account that spasticity is accompanied by one or more symptoms (spasms/cramps, pain, bladder dysfunction, sleep disorders, fatigue and/or tremor). As these symptoms share a common cannabinoid control, therapy acting on cannabinoid receptors may be useful. The main study objectives were to determine the number of MS patients who met Spasticity-Plus syndrome criteria and to identify the most common symptoms.

**Methods:**

Clinical records of MS patients treated with nabiximols in a tertiary hospital from 2002 to 2022 were reviewed retrospectively.

**Results:**

Of the 73 patients included in the study, 53.4% were women, and most had secondary progressive MS (64.4%). All patients met the criteria for Spasticity-Plus syndrome: 100% had spasticity and at least another symptom. Pain was the second most common symptom (91.8%), followed by spasms/cramps (79.4%), and fatigue (76.7%). Sleep disturbances (*p* < 0.0001) and tremor (*p* < 0.027) were more frequent in patients with relapsing–remitting MS than in patients with progressive MS. No statistically significant differences were found for spasticity, pain, spasms/cramps, and fatigue between MS phenotypes. Regarding symptoms clusters, 94.4% of the patients had three or more symptoms. Spasticity was more frequently associated with pain (91.8%) and spasms/cramps (79.4%).

**Conclusion:**

Spasticity-Plus syndrome was present in all the study population of patients with different MS phenotypes, and treated with nabiximols.

## 1 Introduction

Multiple sclerosis (MS) is a common and often disabling neurological disease with a wide variety of symptoms. According to its phenotype, MS can be classified as primary progressive multiple sclerosis (PPMS), secondary progressive multiple sclerosis (SPMS), and relapsing–remitting multiple sclerosis (RRMS). Spasticity is a common MS symptom affecting 60–84% of MS patients (1) and is characterized by hypertone, a velocity-dependent increase of muscle tone. Demyelination at different levels of corticospinal tract causes loss of inhibitory control on spinal tonic stretch reflexes, resulting in spasticity (2, 3). According to a classical model, hypertone triggers a series of clinical manifestations associated with spasticity, with proporcionality between severity of spasticity and that of the other manifestations. However, this model does not totally agree with clinical practice situations (3). Then, another explanation for spasticity and associated symptoms has been suggested (4). When the upper motor neuron in the corticospinal tract is damaged, manifestations can be positive irritative (spasticity) and negative deficient (weakness). Nevertheless, patients can show severe spasticity without significant weakness. Then, it has been proposed another explanation based on the fiber diameters. Larger diameter fibers course along the lateral column of the spinal cord, while smaller fibers are located along the anteromedial column. It has been hypothesized that smaller fibers are more sensitive than larger ones to demyelination, because they are more sensitive to conduction block and more prone to develop ephaptic transmission in front of demyelination (3). According to this theory, some MS symptoms such as spasticity, fatigue, and urinary retention could be derived from conduction block, while others symptoms, such as spasms/cramps, pain, and urinary urgency, could be explained by demyelinated fiber hyperexcitability and secondary ephaptic transmission (3).

Spasticity is often associated with other symptoms, mainly spasms/cramps and pain (5). Awareness of these clusters of symptoms prompted the definition of the Spasticity-Plus syndrome (4), where spasticity occurs together with one or more of the following symptoms: spasms-cramps, pain, bladder dysfunction, sleep disorders, fatigue and tremor (4). The concept of Spasticity-Plus syndrome places all these symptoms on the same level and, therefore, spasticity could be as severe as the other associated symptoms or even milder (3). In addition, there are other symptoms, such as sexual dysfunction (6, 7) and mood disorders (8), that are not under the umbrella of Spasticity-Plus syndrome but are relevant to MS patients and can be associated with spasticity (5, 9).

Muscle tone, pain pathway, bladder function and sleep are controlled by cannabinoid CB_1_ and CB_2_ receptors, that are distributed widely within the CNS, especially in the brainstem, as well as at all levels of the cortical spinal tract (3, 4). Therefore, it has been proposed that activity on these receptors could improve a range of associated MS manifestations (3, 4). Nabiximols, a compound of δ-9-tetrahydrocannabinol (THC) and cannabidiol (CBD) administered as an oromucosal spray, interacts with CB_1_ and CB_2_ receptors (10). Furthermore, data from randomized clinical trials (11, 12) and observational studies (13–15) show the effectiveness of nabiximols in spasticity-associated symptoms. It is approved in Europe, Israel and Canada as add-on therapy for the symptomatic relief in patients with MS and spasticity as add-on therapy for the symptomatic relief in patients with MS and spasticity refractory to standard treatment. However, according to a recent review and meta-analysis, more studies are needed on issues such as duration of treatment or time of onset (16).

The objectives of this study were to determine the number of MS patients treated with nabiximols who met Spasticity-Plus syndrome criteria and to identify the most common symptoms.

## 2 Materials and methods

### 2.1 Study design and population

This was an observational, retrospective study in adult patients diagnosed with MS and managed in an advanced practice nursing clinic within the demyelinating disease unit of a tertiary hospital between January 2002 and December 2022. Patients diagnosed with MS and past or current treatment with nabiximols were included in the study. Patients with any diagnosis different from MS, despite receiving treatment with nabiximols, were excluded. MS was diagnosed using the McDonald 2010 diagnostic criteria.

Information was obtained retrospectively from the electronic medical records of patients, including sex, age, MS phenotype, physical disability [assessed with the Expanded Disability Status Scale (EDSS) (17)], and number of symptomatic treatments at diagnosis and currently. Neurologists searched for spasticity when patients complained about stiffness, rigidity, spasms or other symptoms related to spasticity and only those that met spasticity diagnosis were included in the study. Data regarding present MS-associated symptoms (spasticity, spasms/cramps, pain, sleep disturbances, bladder dysfunction, fatigue and tremor) were also collected. These symptoms were assessed according to the neurologists’ criteria. In addition, the use of symptomatic treatments, including nabiximols, and disease-modifying drugs (DMDs), was recorded. All study information, including pseudo-anonymized personal data, was gathered in a specifically-designed database.

### 2.2 Statistical analyses

A descriptive analysis was performed, with continuous variables expressed as mean ± SD or median and interquartile range, and categorical variables reported as frequency (*n*) and percentage (%). In addition, Venn diagrams were generated to show the clusters of the four most common symptoms (spasticity, pain, spasms/cramps, and fatigue), as well as pain and the three least common symptoms (sleep disturbances, bladder dysfunction and tremor), in all patients and in PPMS/SPMS and RRMS patients. A *p* value <0.05 was considered statistically significant. All analyses were performed using IBM^®^ SPSS^®^ Statistics software version 29.0.

## 3 Results

### 3.1 Whole study population

Of the 73 patients included in the study, 53.4% were women, and most had SPMS (64.4%). Mean EDSS score was 6.5. All patients had spasticity. The second most common symptom was pain (91.8%), followed by spasms/cramps (79.4%), and fatigue (76.7%) (Table 1).

**Table 1:** Baseline demographic and clinical characteristics.

Variable	Population (*n* = 73)
Female sex, *n* (%)	39 (53.4)
MS phenotype, *n* (%)	
Primary progressive MS	14 (19.2)
Relapsing–remitting MS	12 (16.4)
Secondary progressive MS	47 (64.4)
EDSS score, median (IQR)	6.5 (1.5)
MS-associated symptoms, *n* (%)	
Spasticity	73 (100)
Pain	67 (91.8)
Spasms/cramps	62 (85.0)
Fatigue	58 (79.5)
Bladder dysfunction	32 (43.9)
Tremor	28 (38.4)
Sleep disturbances	22 (30.1)
Number of symptomatic drugs, mean ± SD	
At baseline	1.5 ± 1.7
Currently	3.9 ± 2.5

MS, multiple sclerosis; EDSS, expanded disability status scale; IQR, interquartile range.

Regarding symptomatic treatment, the number of drugs used increased with time, from 1.51 ± 1.72 drugs at diagnosis to 3.86 ± 2.49 drugs currently. All patients received or had received nabiximols. Furthermore, 34 patients (46.6%) were currently being treated with DMDs, mainly rituximab (6 patients), azathioprine (6 patients), and ocrelizumab (5 patients).

### 3.2 Results by MS course type

**Figure 1:**
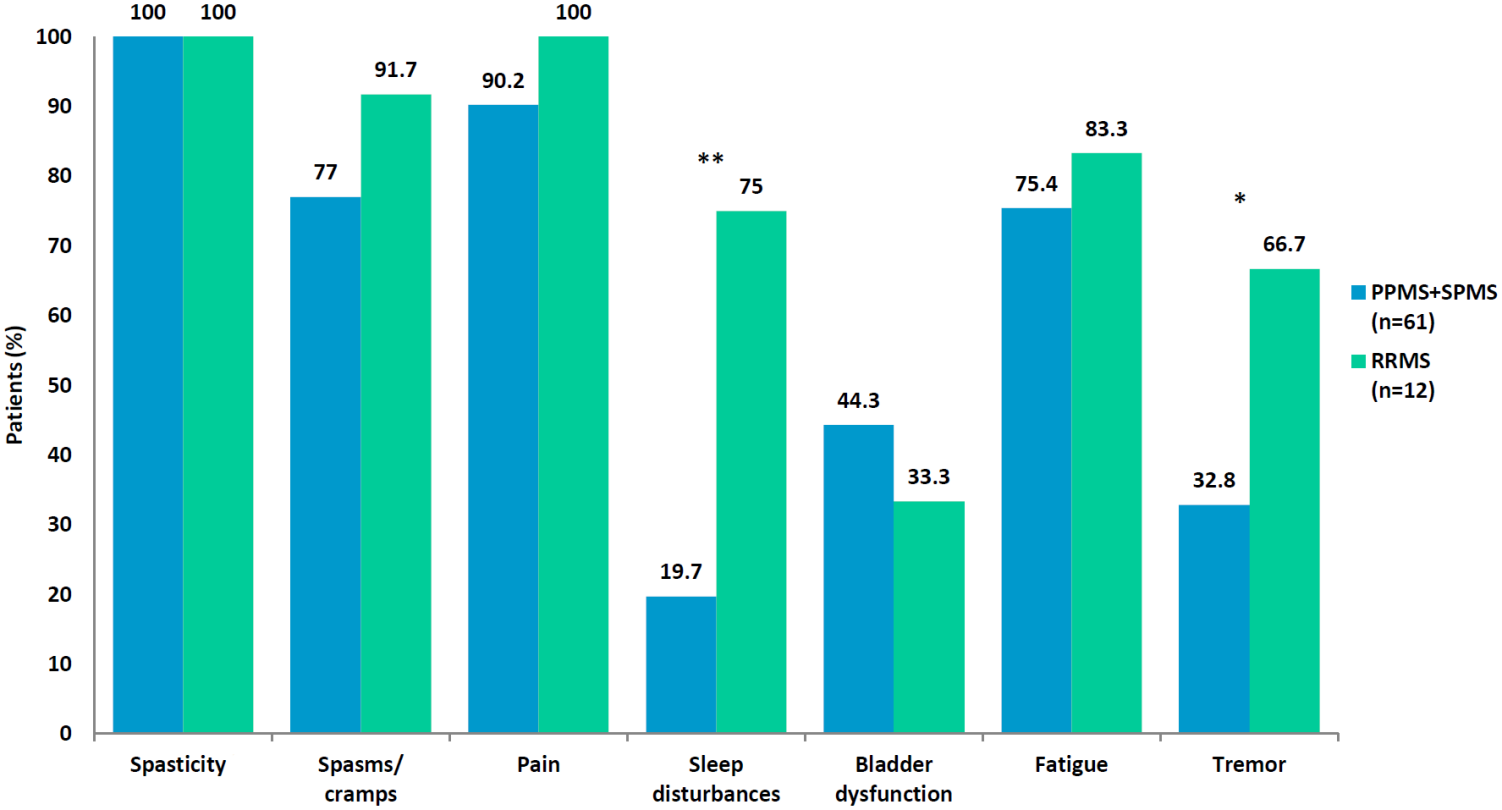
Spasticity and associated symptoms in 73 patients with multiple sclerosis (MS) according to MS phenotype. *p < 0.027; **p < 0.0001. PPMS, primary progressive MS; SPMS, secondary progressive MS; RRMS, relapsing–remitting MS.

The frequency of MS-associated symptoms was analyzed in PPMS/SPMS and RRMS patients. Regardless of their phenotype, all patients had spasticity. Sleep disturbances (*p* < 0.0001) and tremor (*p* < 0.027) were more common in patients with RRMS, while no statistically significant differences were found for spasticity (*p* = 1), pain (*p* = 0.257), spasms/cramps (*p* = 0.252), bladder dysfunction (*p* = 0.484), and fatigue (*p* = 0.553) (Figure 1).

### 3.3 Clusters of symptoms

**Figure 2:**
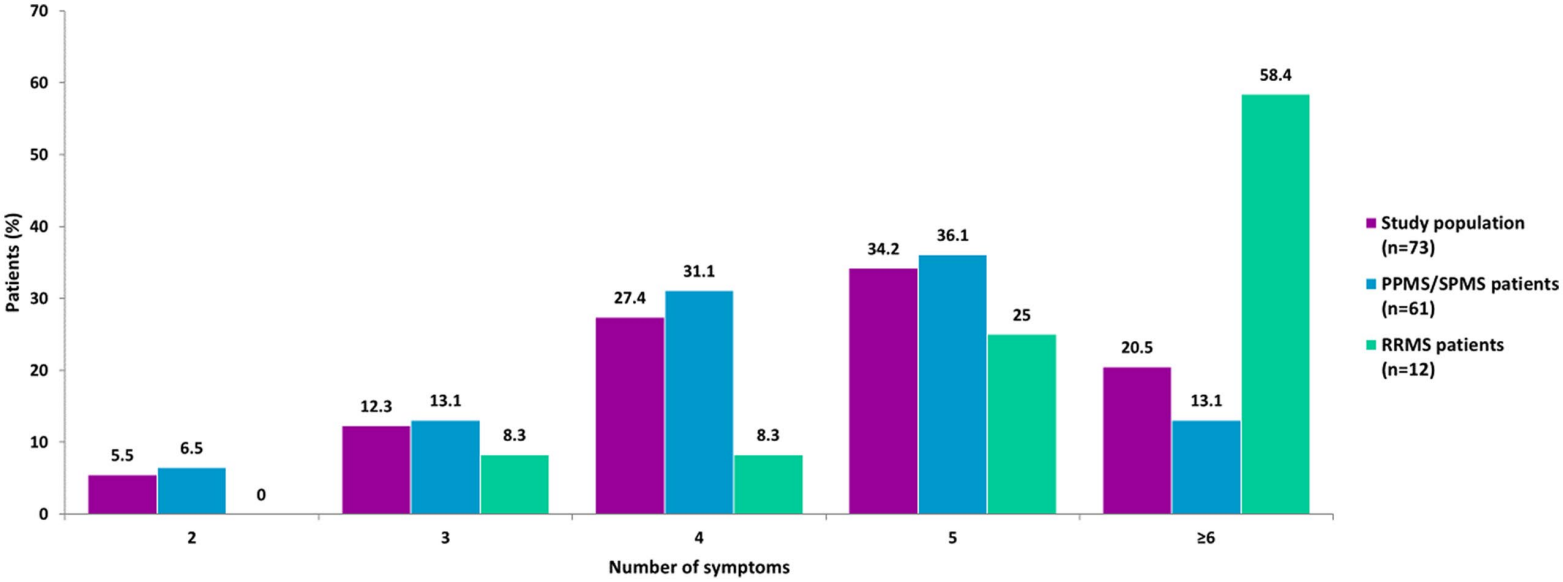
Number of symptoms by MS phenotypes. Three patients (1 with PPMS and 2 with RRMS) had all seven symptoms. No patient had only 1 symptom. PPMS, primary progressive MS; SPMS, secondary progressive MS; RRMS, relapsing–remitting MS.

No patient had only spasticity or just one MS-associated symptom. Almost all patients (98.5%) had three or more symptoms and 50.2% had five or more symptoms. Three patients (1 with PPMS and 2 with RRMS, 4.1%) had all seven symptoms. Patients with progressive phenotypes most frequently had four or five symptoms (67.2%), while patients with RRMS had five or more symptoms (83.4%) (Figure 2).

Venn diagrams were generated to show the clusters of symptoms for the four most common symptoms (spasticity, pain, spasms/cramps, and fatigue), as well as pain and the three least common symptoms (bladder dysfunction, sleep disturbances, and tremor) for all patients, and for PPMS/SPMS and RRMS patients.

**Figure 3:**
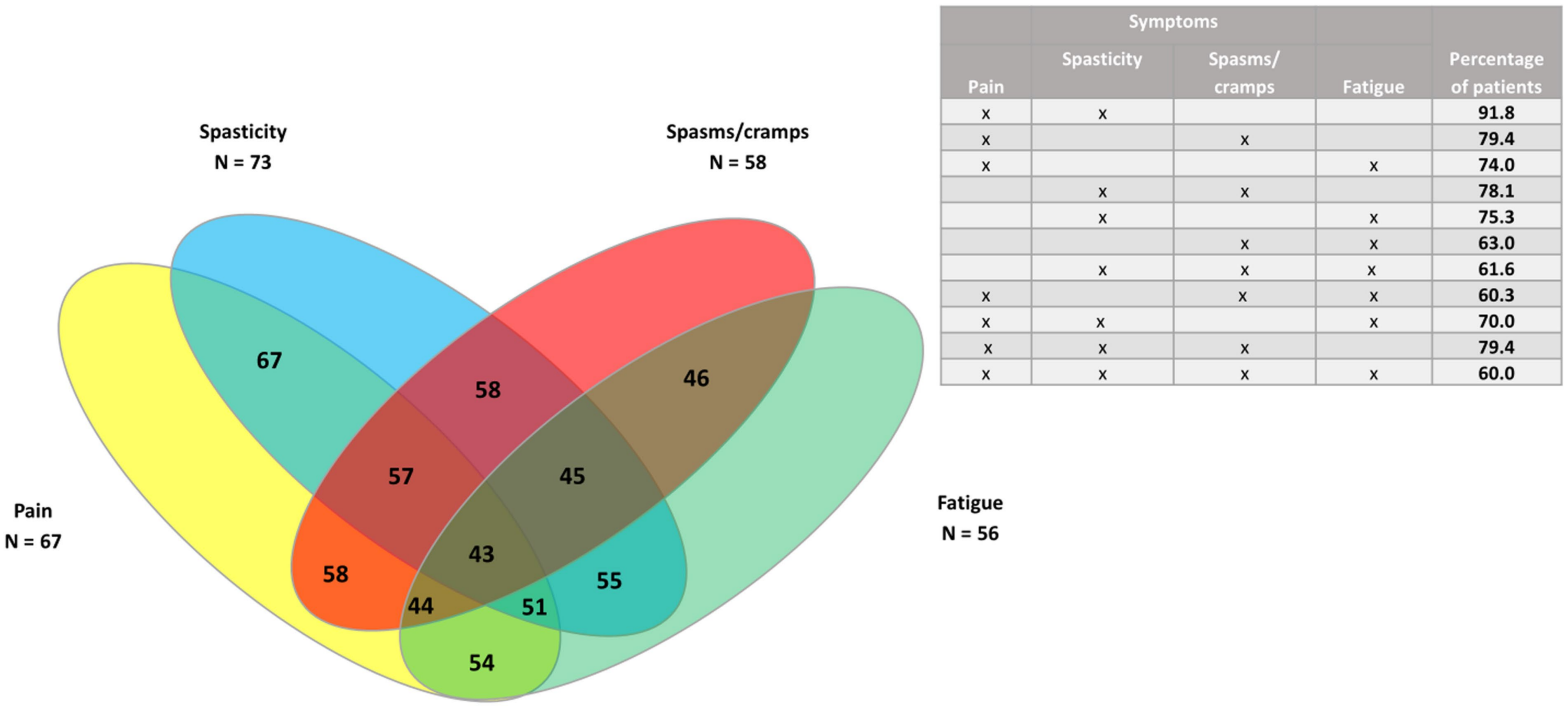
Venn diagram of the four most common symptoms (spasticity, pain, spasms/cramps, and fatigue) in the whole study population (*n* = 73).

**Figure 4:**
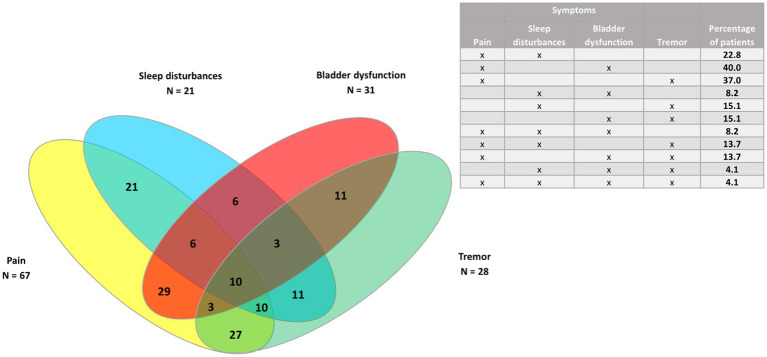
Venn diagram of pain and the three least common symptoms (bladder dysfunction, sleep disturbances, and tremor) in the whole study population (*n* = 73).

In the whole study population (*n* = 73) (Figures 3, 4), the largest clusters were spasticity and pain (91.8%); pain and spasms/cramps (79.4%); spasticity and spasms/cramps (78.1%); pain and fatigue (74%). The most common cluster of three symptoms was spasticity, spasms/cramps, and pain (79.4%) and the most common cluster of four symptoms was spasticity, spasms/cramps, pain, and fatigue (60%).

**Figure 5:**
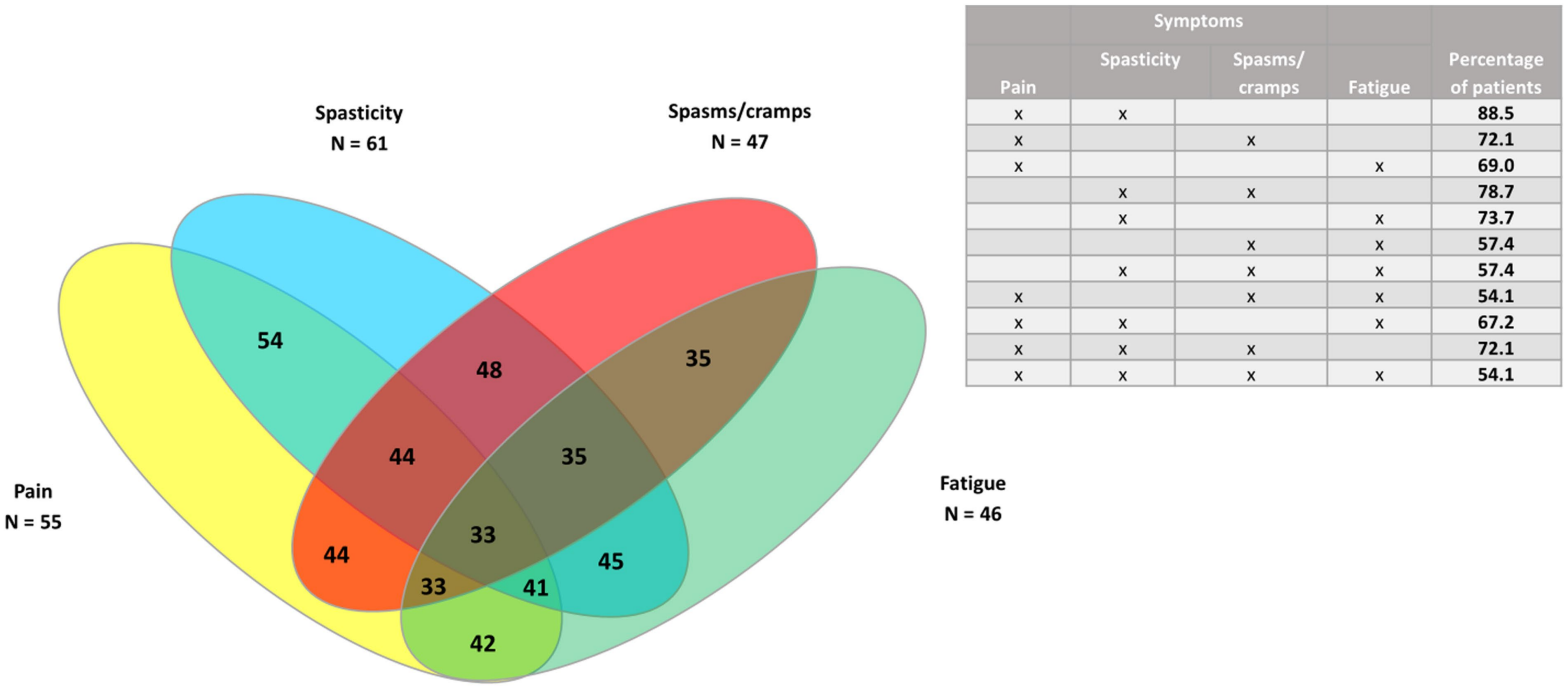
Venn diagram of the four most common symptoms (spasticity, pain, spasms/cramps, and fatigue) in patients with progressive MS phenotypes (*n* = 61).

**Figure 6:**
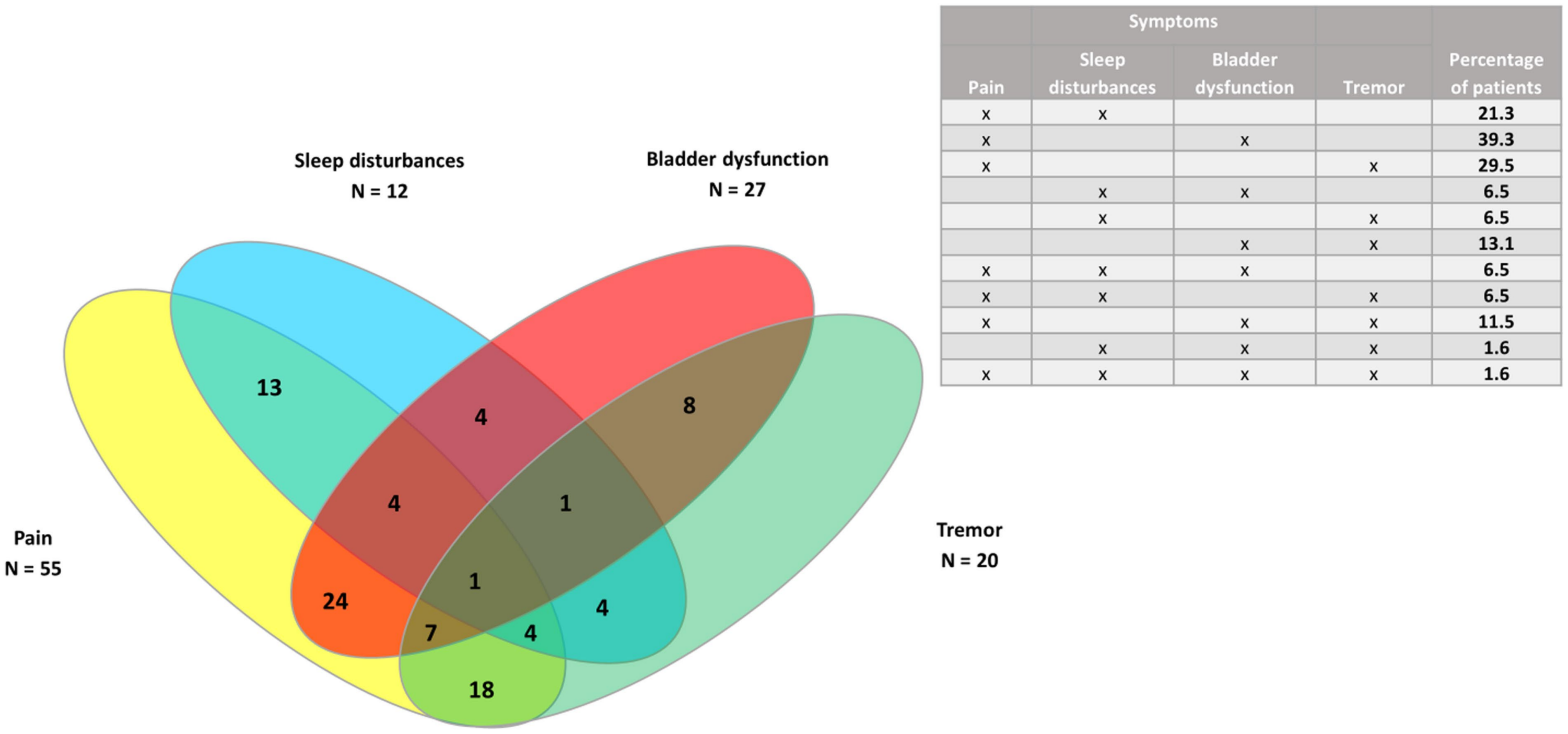
Venn diagram of pain and the three least common symptoms (bladder dysfunction, sleep disturbances, and tremor) in patients with progressive MS phenotypes (*n* = 61).

In the 61 patients with progressive MS phenotypes (Figures 5, 6), the largest clusters were spasticity and pain (88.5%); spasticity and spasms/cramps (78.7%); spasticity and fatigue (73.7%); spasms/cramps and pain (72.1%), and spasticity, spasms/cramps and pain (72.1%).

**Figure 7:**
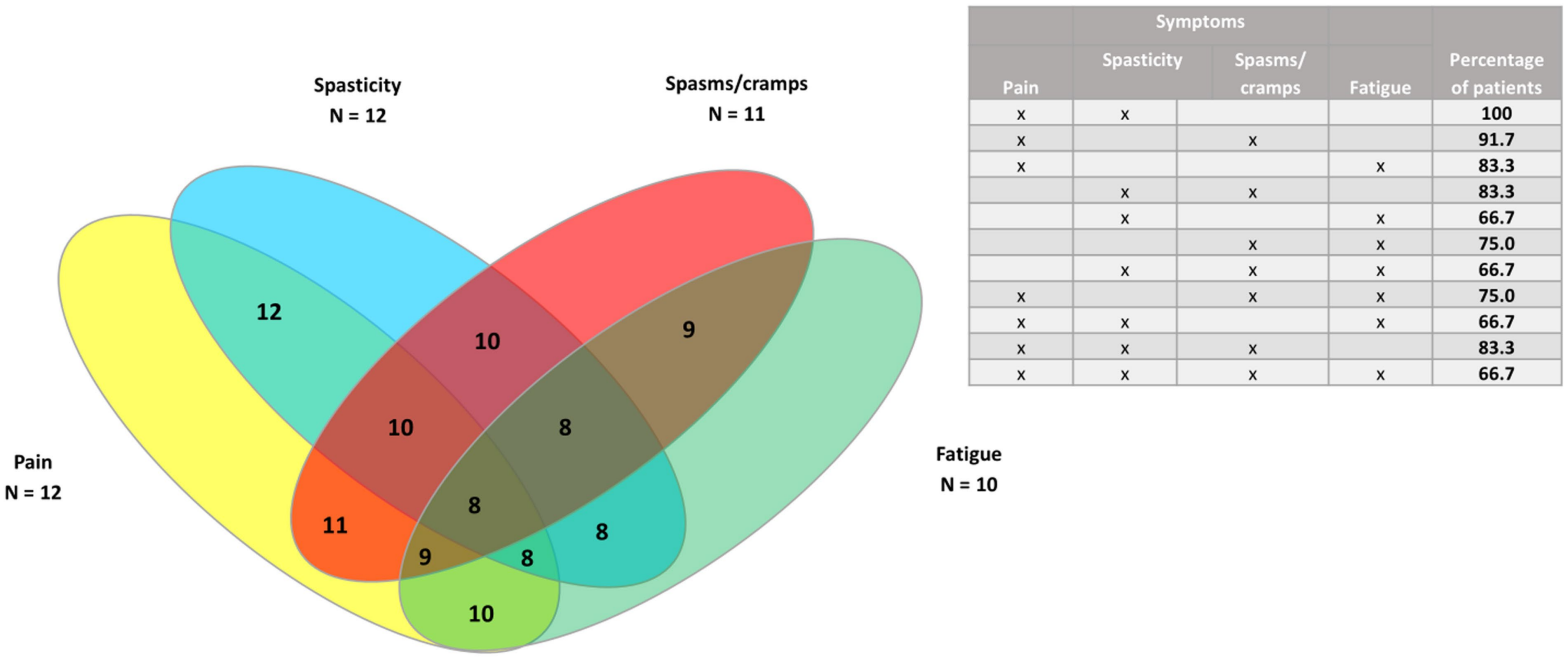
Venn diagram of the four most common symptoms (pain, spasms/cramps, spasticity, and fatigue) in patients with relapsing–remitting MS (*n* = 12).

**Figure 8:**
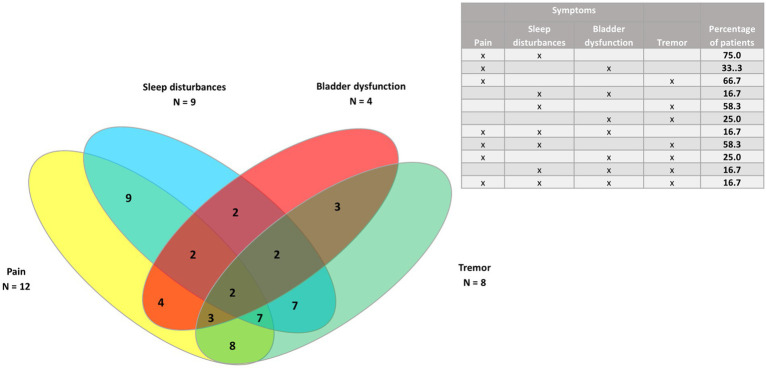
Venn diagram of pain and the three least common symptoms (sleep disturbances, tremor, and bladder dysfunction) in patients with relapsing–remitting MS (*n* = 12).

In the 12 patients with RRMS (Figures 7, 8), all had spasticity and pain. Other common clusters were pain and spasms/cramps (91.7%); spasticity and spasms/cramps; pain and fatigue; spasticity, spasms/cramps, and pain (83.3% for each cluster).

## 4 Discussion

This retrospective study showed that Spasticity-Plus syndrome was ubiquitous in a population of MS patients treated with nabiximols. Patients were mainly women, although the female/male ratio was lower than that reported for Spain and Europe (18), and had significant physical disability (mean EDSS score of 6.5). The most common MS phenotype was SPMS (65%), as reported in other studies (14, 15, 19). The mean number of symptomatic treatments used by patients increased over time.

All the patients included in the study had spasticity independently of their MS phenotype. Spasticity was not an inclusion criterion, but all the patients had it because nabiximols is indicated in Europe for the treatment of this symptom in MS patients refractory to other treatments. Furthermore, all the patients met the criteria for Spasticity-Plus syndrome, because they had at least one spasticity-associated symptom. The second most common symptom was pain, followed by spasms/cramps, and fatigue. Bladder dysfunction, tremor and sleep disturbances were less common, but still present in almost one-third of patients. No patient had just one symptom, either spasticity or a spasticity-associated symptom; this finding highlights the clustering of symptoms in MS and aligns with the concept of Spasticity-Plus syndrome.

Regarding symptoms by phenotype, pain was the second most common symptom in both PPMS/SPMS and RRMS patients. Sleep disturbances and tremor were more frequent in RRMS patients. No other statistically significant differences were found. Furthermore, RRMS patients had more clustered symptoms. We did not find other studies reporting such differences. However, we did not analyze the potential reasons for these differences because of the low number of RRMS patients in the study.

Regarding clustering of symptoms, all the patients had spasticity and at least another symptom. The largest clusters were of spasticity, spasms/cramps and pain, and the cluster of those three symptoms plus fatigue. Pain was the symptom most frequently associated with spasticity, as also found in other studies (14, 20). In this study, pain was more common in RRMS patients but the difference was not statistically significant. Chronic pain is very prevalent and heterogeneous in patients with MS. However, the effects of analgesic medications are poor to moderate (21). There is not a single type of pain in MS and many different types have been suggested (22), including neuropathic pain (23) such as trigeminal neuralgia (14). Therefore, pain assessment in MS patients should be multidimensional in order to choose the best treatment (21) as well as using the fewest number of drugs to avoid polytherapy.

It has been suggested that when a patient with MS has one or more spasticity-associated symptoms, physicians should evaluate the presence of other symptoms (4). However, the finding of another symptom may involve the prescription of additional medication to a possibly polymedicated patient. Simplifying the treatment of spasticity and associated symptoms could help to avoid the adverse events and drug-to-drug reactions that can occur with commonly used anti-spasticity drugs (5). Within the framework of the Spasticity-Plus syndrome, using a single treatment that acts on the cannabinoid system may improve more than one symptom (4). The effect of therapies, including nabiximols, on the symptoms of Spasticity-Plus syndrome was not assessed because it was not an objective of the current study.

However, a recent systematic review and meta-analysis of randomized clinical trials of data from 1,128 MS patients showed the efficiency of nabiximols as add-on therapy in spasticity that is refractory to standard treatment (16). It would have been interesting to analyze the potential improvement of spasticity and other symptoms after nabiximols or other therapies in the study population, as it would mirror the real-world situation. According to an expert panel, nabiximols not only improves spasticity, but it also demonstrates adequate results for other symptoms such as pain, spasms/cramps, spastic bladder and, in some cases, sleep disturbances (5). In real-world observational studies, spasticity and associated symptoms have improved after nabiximols treatment (14, 15). These effects on spasticity-associated symptoms were independent of spasticity reduction and reinforced the concept of Spasticity-Plus syndrome (14).

The main strength of the current study is that the results could be useful for improving the early diagnosis of Spasticity-Plus syndrome and the initiation of a specific therapy as soon as possible, as well as avoiding polytherapy. Furthermore, this study has some limitations. It was a retrospective cohort and it could be a selection bias. Patients were only included if they received past or current nabiximols treatment and, therefore, some patients with MS and spasticity but not receiving nabiximols could have been missed. Another limitation was the low number of patients with RRMS. This makes sense because progressive forms are associated with more symptoms than RRMS (24). A third limitation was that we did not have data of nabiximols efficacy and safety despite all the patients were or had been treated with this drug.

In conclusion, presence of Spasticity-Plus syndrome was total in a population of MS patients treated with nabiximols, both in progressive and relapsing–remitting MS forms. This result can help to early diagnose this syndrome. Pain was the most common symptom associated to spasticity, followed by spasms/cramps, in all the MS forms. However, sleep disturbances and tremor were more frequent in RRMS patients. Using a treatment that acts both on spasticity and associated symptoms could simplify MS management.

## Data availability statement

The raw data supporting the conclusions of this article will be made available by the authors, without undue reservation.

## Ethics statement

The studies involving humans were approved by the Medical Research Ethics Committee of Area 1 of the Gregorio Marañón University General Hospital, Madrid, Spain. Patients provided written informed consent to participate in this study. The studies were conducted in accordance with the local legislation and institutional requirements. The participants provided their written informed consent to participate in this study.

## Author contributions

HG: Writing – original draft, Writing – review & editing. YH: Writing – original draft, Writing – review & editing. IR: Writing – original draft, Writing – review & editing. JG: Writing – original draft, Writing – review & editing. JC: Writing – original draft, Writing – review & editing. AM: Writing – original draft, Writing – review & editing. MM: Writing – original draft, Writing – review & editing.
